# Nonenhanced hybridized arterial spin labeled magnetic resonance angiography of the extracranial carotid arteries using a fast low angle shot readout at 3 Tesla

**DOI:** 10.1186/s12968-016-0238-1

**Published:** 2016-04-12

**Authors:** Ioannis Koktzoglou, Matthew T. Walker, Joel R. Meyer, Ian G. Murphy, Robert R. Edelman

**Affiliations:** Department of Radiology, NorthShore University HealthSystem, Evanston, USA; University of Chicago Pritzker School of Medicine, Chicago, USA; Northwestern University Feinberg School of Medicine, Chicago, USA

**Keywords:** Magnetic resonance angiography, Carotid, Intracranial, Arterial spin labeling, Nonenhanced

## Abstract

**Background:**

To evaluate ungated nonenhanced hybridized arterial spin labeling (hASL) magnetic resonance angiography (MRA) of the extracranial carotid arteries using a fast low angle shot (FLASH) readout at 3 Tesla.

**Methods:**

In this retrospective, institutional review board-approved and HIPAA-compliant study, we evaluated the image quality (4-point scale) of nonenhanced hASL MRA using a FLASH readout with respect to contrast-enhanced MRA (CEMRA) in 37 patients presenting with neurologic symptoms. Two certified neuroradiologists independently evaluated 407 arterial segments (11 per patient) for image quality. The presence of vascular pathology was determined by consensus reading. Gwet’s AC1 was used to assess inter-rater agreement in image quality scores, and image quality scores were correlated with age and body mass index. Objective measurements of arterial lumen area and sharpness in the carotid arteries were compared to values obtained with CEMRA. Comparisons were also made with conventional nonenhanced 2D time-of-flight (TOF) MRA.

**Results:**

CEMRA provided the best image quality, while nonenhanced hASL FLASH MRA provided image quality that exceeded 2D TOF at the carotid bifurcation and in the internal and external carotid arteries. All nine vascular abnormalities of the carotid and intracranial arteries detected by CEMRA were depicted with hASL MRA, with no false positives. Inter-rater agreement of image quality scores was highest for CEMRA (AC1 = 0.87), followed by hASL (AC1 = 0.61) and TOF (AC1 = 0.43) (*P* < 0.001, all comparisons). With respect to CEMRA, agreement in cross-sectional lumen area was significantly better with hASL than TOF in the common carotid artery (intraclass correlation (ICC) = 0.90 versus 0.66; *P* < 0.05) and at the carotid bifurcation (ICC = 0.87 versus 0.54; *P* < 0.05). Nonenhanced hASL MRA provided superior arterial sharpness with respect to CEMRA and 2D TOF (*P* < 0.001).

**Conclusion:**

Although inferior to CEMRA in terms of image quality and inter-rater agreement, hASL FLASH MRA offers an alternative to 2D TOF for the nonenhanced evaluation of the extracranial carotid arteries at 3 Tesla. Compared with 2D TOF, nonenhanced hASL FLASH MRA provides improved quantification of arterial cross-sectional area, vessel sharpness, inter-rater agreement and image quality.

## Background

Disorders of the extracranial carotid arteries including stenoses, dissections and aneurysms are frequently evaluated using contrast-enhanced magnetic resonance angiography (CEMRA). However, CEMRA is contraindicated in patients with moderate to severe renal insufficiency, which can be present in over 25 % of patients with stroke [1–4]. Furthermore, in patients with suspected stroke, contrast agents may be reserved for the assessment of cerebral perfusion [5].

Nonenhanced MRA may address the above drawbacks of CEMRA. Time-of-flight (TOF) magnetic resonance angiography (MRA) is a well-established and easy-to-use method for diagnosing disorders of the extracranial carotid arteries without the use of contrast agents [6–10]. Although it is often used in clinical practice, TOF has well-known drawbacks including artifacts from saturation and dephasing of flowing spins, as well as limited vascular coverage with respect to CEMRA [11]. To address the shortcomings of TOF, as well as to potentially better serve patients with renal insufficiency, alternate nonenhanced MRA techniques have been reported, including inversion-recovery fast spin-echo or balanced steady-state free precession (bSSFP) angiography [12–14], and quiescent-interval slice-selective (QISS) angiography using a fast low-angle shot (FLASH) readout [15]. Raoult and colleagues [13] postulated that better suppression of static background tissue would likely improve the clinical utility of nonenhanced MRA of the extracranial carotid arteries, and mentioned that arterial spin labeling (ASL) methods, initially described long ago [16, 17], might achieve this.

Recent work has reported the potential of advanced ASL-based MRA for imaging arteries of the head and neck with complete suppression of static background signal [18–20]. In particular, a hybrid of pseudo-continuous and pulsed ASL (hASL) has been shown to efficiently portray long lengths of the extracranial carotid arteries at 1.5 Tesla without the need for cardiac gating [21], with a FLASH variant providing the most accurate portrayal of stenoses in vitro [22]. On the basis of these reports, our department incorporated an ungated 3D FLASH variant of hASL MRA into the standard-of-care neck MRA exam at 3 Tesla to serve as a pre-contrast scout for clinical purposes only. The purpose of this retrospective study was to evaluate the image quality of this clinical scout protocol for portraying the extracranial carotid arteries at 3 Tesla in patients undergoing 2D TOF and CEMRA protocols.

## Methods

In this retrospective, Health Insurance Portability and Accountability Act-compliant study, a waiver of the requirement for patient consent was approved by the institutional review board of NorthShore University HealthSystem. Nonenhanced 3D hASL MRA was acquired as a clinical scout scan prior to injection of contrast media in consecutive patients who were referred for neck MRA with and without contrast material.

Study inclusion criteria were age ≥18 years and referral based on the clinical suspicion of stenosis or stroke and evaluation with TOF, hASL MRA, and CEMRA. Study exclusion criteria included the following: contraindications to cardiovascular magnetic resonance (CMR), renal impairment that precluded CEMRA (defined by glomerular filtration rate lower than 30 mL/min/1.73 m^2^), inability to complete or non-diagnostic image quality on any of the three MRA acquisitions (hASL, TOF, CEMRA), and previous arterial revascularization including stent placement. No scans were re-acquired in the case of non-diagnostic image quality.

### Imaging system and protocols

Imaging was performed on a 3 Tesla CMR system (MAGNETOM Skyra, Siemens Healthcare, Erlangen, Germany) having a maximum gradient strength of 45mT/m and a maximum slew rate of 200mT/m/ms. The CMR signal was received by a 20-channel head and neck coil (Head/Neck 20, Siemens Healthcare, Erlangen, Germany). Imaging was performed with 2D TOF MRA, 3D hASL MRA, and 3D CEMRA. TOF and CEMRA were acquired using institutional standard-of-care protocols. CEMRA was performed using 0.1 mmol/kg of gadobutrol (Gadavist, Bayer HealthCare, Whippany, NJ) injected in an antecubital vein at 2 mL/s. Imaging parameters for all protocols are listed in Table 1.

**Table 1:** Imaging Parameters

	hASL	TOF	CEMRA
Orientation	coronal	axial	coronal
Acquisition type	3D	2D	3D
TR (ms)	5.8	19.0	3.2
TE (ms)	3.9	3.7	1.2
Flip angle (degrees)	5	60	25
Field of view (mm)^a^	256 × 256	220 × 220	320 × 260
	[256-320 × 256-320]		
Matrix	256 × 256	256 × 256	352 × 286
Slices^a^	60 [60–80]	100 [60-120]	80
In-plane resolution (mm)^a^	1.0 × 1.0	0.9 × 0.9	0.9 × 0.9
	[1.0-1.25 × 1.0-1.25]		
Slice thickness (mm)	1.0	2.0	1.2
Partial Fourier (phase)	none	none	6/8th
Partial Fourier (slice)	6/8th	none	6/8th
Scan time^a^	4.6 [4.6–6.1] min	4.7 [2.8–5.6] min	19 s
Flow Compensation	yes	yes	no
Slice Oversampling	none	--	20 %
Bandwidth (Hz/pixel)	349	465	590

^a^values given as median [range]; all protocols used a generalized auto-calibrating partially parallel acquisition (GRAPPA) factor of 2

**Fig. 1:**
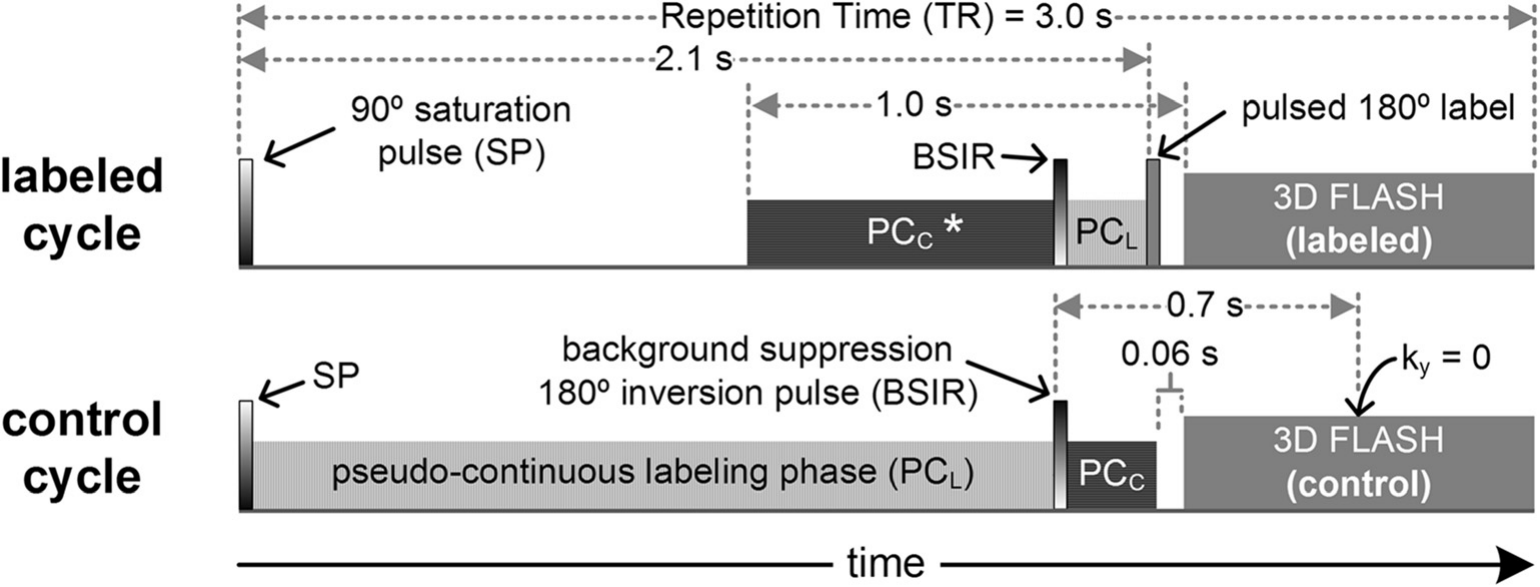
Timing diagram of the hASL MRA protocol. The “labeled cycle” (*top panel*) and the “control cycle” (*bottom panel*) were acquired in an interleaved manner. Using a parallel acceleration factor of 2, 140 phase-encoding steps were acquired in each cycle. The sequence repeated until all slice-encoding steps were collected. Complex subtraction of data acquired in the two readouts produced the angiogram. Pseudo-continuous (PC) RF labeling (1 cm thickness), pulsed RF labeling (10 cm thickness) and an inversion RF pulse for background suppression (BSIR) (20 cm thickness) were applied 5 cm below, 10 cm below and 5 cm above the center of the coronal imaging slab, respectively. Parameters for pseudo-continuous labeling were: 1.5 ms repetition time, 25° flip angle, 750 μs RF duration, 3mT/m maximum gradient strength, 0.5mT/m average gradient strength. The axial 10 cm-thick pulsed RF inversion was applied 60 ms before the fast low-angle shot (FLASH) readout. An abbreviated pseudo-continuous control phase (PC_C_) indicated by the asterisk (*) was used during the “labeled cycle” to lessen RF power deposition and neutralize magnetization transfer effects. PC_L_ = pseudo-continuous labeling phase; TR = repetition time; k_y_ = 0 denotes central phase-encoding line

hASL MRA consisted of a prototype ungated 3D coronal FLASH readout (Fig. 1) that was preceded by pseudo-continuous [23] and pulsed [16] radiofrequency (RF) labeling; the timing of the sequence was similar to that of a prior report [21] with minor differences to account for the use of a FLASH readout (see Fig. 1 caption for details). Locations of RF labeling planes were transparent to the CMR operator; the operator positioned the coronal slab over the carotid arteries (centering at the approximate level of the carotid bifurcations) and executed the scan.

### Qualitative analysis

After data acquisition, image processing was performed on a workstation (Leonardo; Siemens Healthcare, Erlangen, Germany) by a CMR scientist (I.K.) who did not participate in image scoring. After non-vascular background tissue was cropped using a 3D volume visualization and editing tool, rotating maximum intensity projection (MIP) image sets (72 projections separated by 5°) were created from each MR angiographic volume. These image sets were anonymized, randomized and then independently reviewed by two certified neuroradiologists (M.W. and J.M.). The neuroradiologists were blinded to the patient name, the clinical history of the patient, and the results of other diagnostic procedures.

Image quality was scored for the following 11 locations: 1 and 2 - bilateral common carotid arteries; 3 and 4 - bilateral carotid bulb and proximal internal carotid arteries; 5 and 6 - bilateral mid-cervical internal carotid arteries; 7 and 8 - bilateral petrous internal carotid arteries; 9 and 10 - bilateral external carotid arteries; and 11 - intracranial arteries. The following 4-point scoring system was used: 1 = non-diagnostic, barely visible lumen rendering the segment non-diagnostic; 2 = fair, ill-defined vessel borders with suboptimal image quality for diagnosis; 3 = good, with some minor inhomogeneities not influencing vessel delineation; and 4 = excellent, sharply defined arterial borders with excellent image quality for highly confident diagnosis.

The presence of arterial pathology was noted independently by both reviewers; discrepancies were settled by consensus review.

### Quantitative analysis

**Fig. 2:**
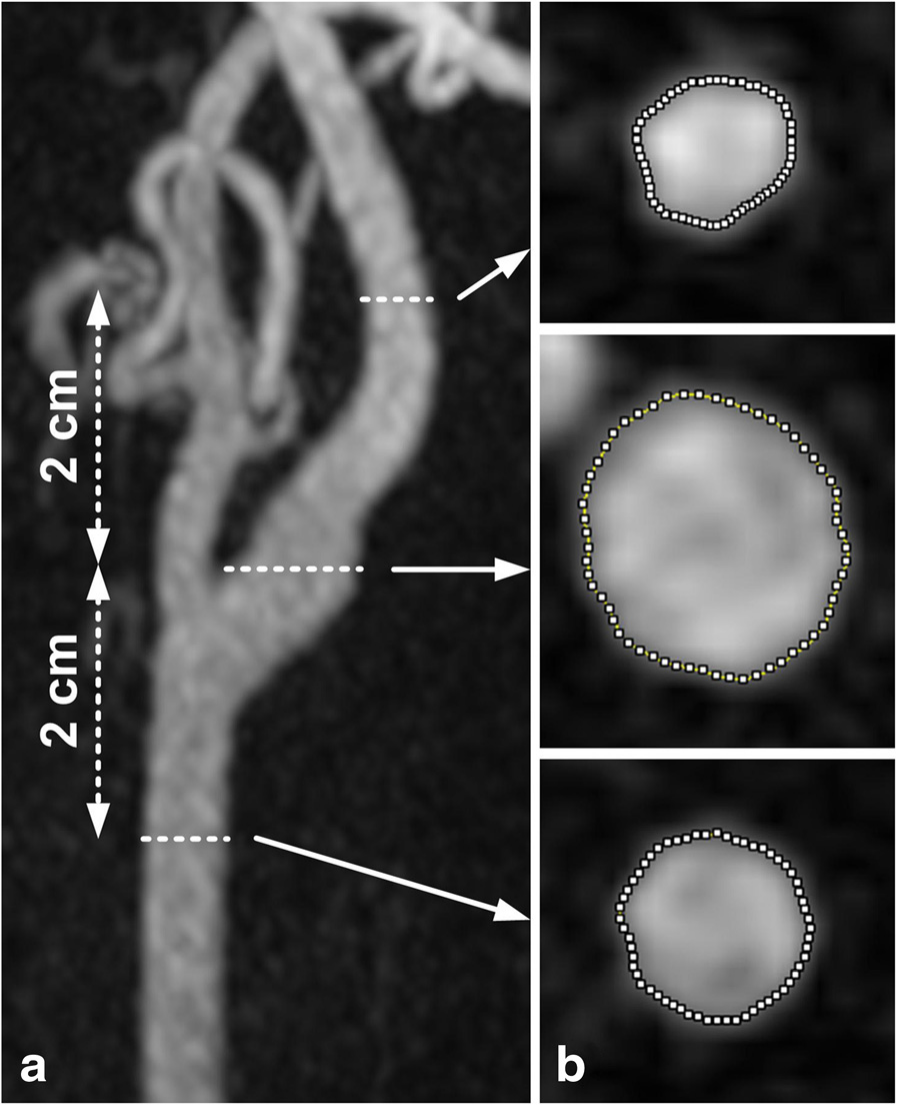
Nonenhanced hASL MRA obtained in a 65-year-old male showing (**a**) the three axial locations (*dashed lines*) where cross-sectional area was measured, and (**b**) luminal contours obtained using objective, full-width-at-half-maximum signal analysis

In each patient, measurements of arterial cross-sectional area and arterial sharpness were obtained in one randomly selected artery per subject, similar to the approach of Kramer et al. [12]. Source images were loaded into 3D image analysis software (Leonardo, Siemens Healthcare, Erlangen, Germany) where axial source reformations were created at three locations (Fig. 2a): location 1 - at the level of the flow divider of the carotid bifurcation, location 2 - common carotid artery two centimeters below location 1, and location 3 - two centimeters above location 1 through the proximal internal carotid artery. Cross-sectional measurements of arterial lumen area for hASL and TOF were compared to CEMRA, which served as the reference standard. Arterial lumen area measurements were obtained in an objective manner by computing the area enclosed by the full-width-at-half-maximum signal points of 60 radial spokes emanating from the center of the vessel (Fig. 2b) [24]. Arterial sharpness at the carotid bifurcation (location 1) was measured as the inverse of the distance between the 20th and 80th percentile points in the 60 spokes [25]; the median sharpness value across the 60 spokes was used.

### Statistical analysis of data

Statistical analysis was performed using R software (version 3.2.1, The R Foundation for Statistical Computing, Vienna, Austria). In handling missing scoring data which occurred if an arterial location was outside the imaged field of view, list-wise deletion was used for comparisons involving three groups while pair-wise deletion was used for comparisons involving two groups.

Due to the inability to conduct the quantitative and diagnostic analyses above, and because no attempts were made to re-acquire scans with non-diagnostic image quality, patients with at least one imaging scan having non-diagnostic image quality (as assessed by median image quality of <2 across 11 segments by at least one reviewer) were excluded from further analysis. Excluded cases were reviewed by a CMR scientist (I.K.) to determine the cause of artifact. In the remaining data, differences in image quality scores between techniques were identified using non-parametric Friedman tests and post-hoc Wilcoxon signed-rank tests. Inter-rater agreement was computed using Gwet’s AC1, which is more reliable than Cohen’s κ when there is a high level of agreement [26]. AC1 was interpreted as follows: 0.01–0.20, slight agreement; 0.21–0.40, fair agreement, 0.41–0.60, moderate agreement; 0.61–0.80, substantial agreement; and 0.81–0.99, almost perfect agreement [27]. The spearman rank correlation coefficient (ρ) was used to evaluate whether image quality (as summarized by the median image quality score across 11 arterial locations and both reviewers) was correlated with age and body mass index (BMI). Agreement of quantitative cross-sectional arterial area measurements was assessed by intraclass correlation coefficient (ICC) and Bland-Altman analysis [28]. Differences in arterial sharpness between techniques were determined using repeated measures analysis of variance with post-hoc Tukey testing. Differences in proportions of diagnostic scans were assessed using a 3-sample test for equality of proportions. P-values less than 0.05 indicated statistical significance.

## Results

Between October 2014 and January 2015, 45 patients underwent MRA of their carotid arteries using hASL, TOF and CEMRA in the same scan session. Eight patients were excluded from analysis due to non-diagnostic studies (median image quality of <2) on either hASL (*n* = 5) and TOF (*n* = 3) exams, leaving data from 37 patients (13 men, 24 women; mean age, 67.5 ± 15.7 years) which were included in our analysis. The percentage of scans with diagnostic image quality (i.e. median image quality scores of ≥2 by both reviewers) was 88.9 % (40/45) for hASL, 93.3 % (42/45) for TOF, and 100 % (45/45) for CEMRA (*P* = NS between techniques). All eight non-diagnostic scans were attributed to motion artifact. Indications for imaging in the remaining 37 patients included dysphasia (*n* = 8), dizziness (*n* = 6), weakness (*n* = 4), transient ischemic attack (*n* = 3), headaches (*n* = 2), visual field deficit (*n* = 2), amnesia (*n* = 2), confusion (*n* = 2), diplopia (*n* = 2), pulsatile tinnitus (*n* = 2), suspected carotid dissection (*n* = 1), aneurysm (*n* = 1), infarct (*n* = 1) and vertigo (*n* = 1).

### Qualitative analysis: summary and segmental analysis

A total of 407 arterial locations (37 subjects, 11 locations/subject) were analyzed. Excluding arterial locations outside the field of view (3 of 407 for CEMRA, 14 of 407 for hASL MRA, and 89 of 407 for TOF MRA), a total of 318 locations were portrayed by all three techniques. Each of these 318 locations was interpreted by two reviewers, resulting in a total of 636 evaluations.

**Fig. 3:**
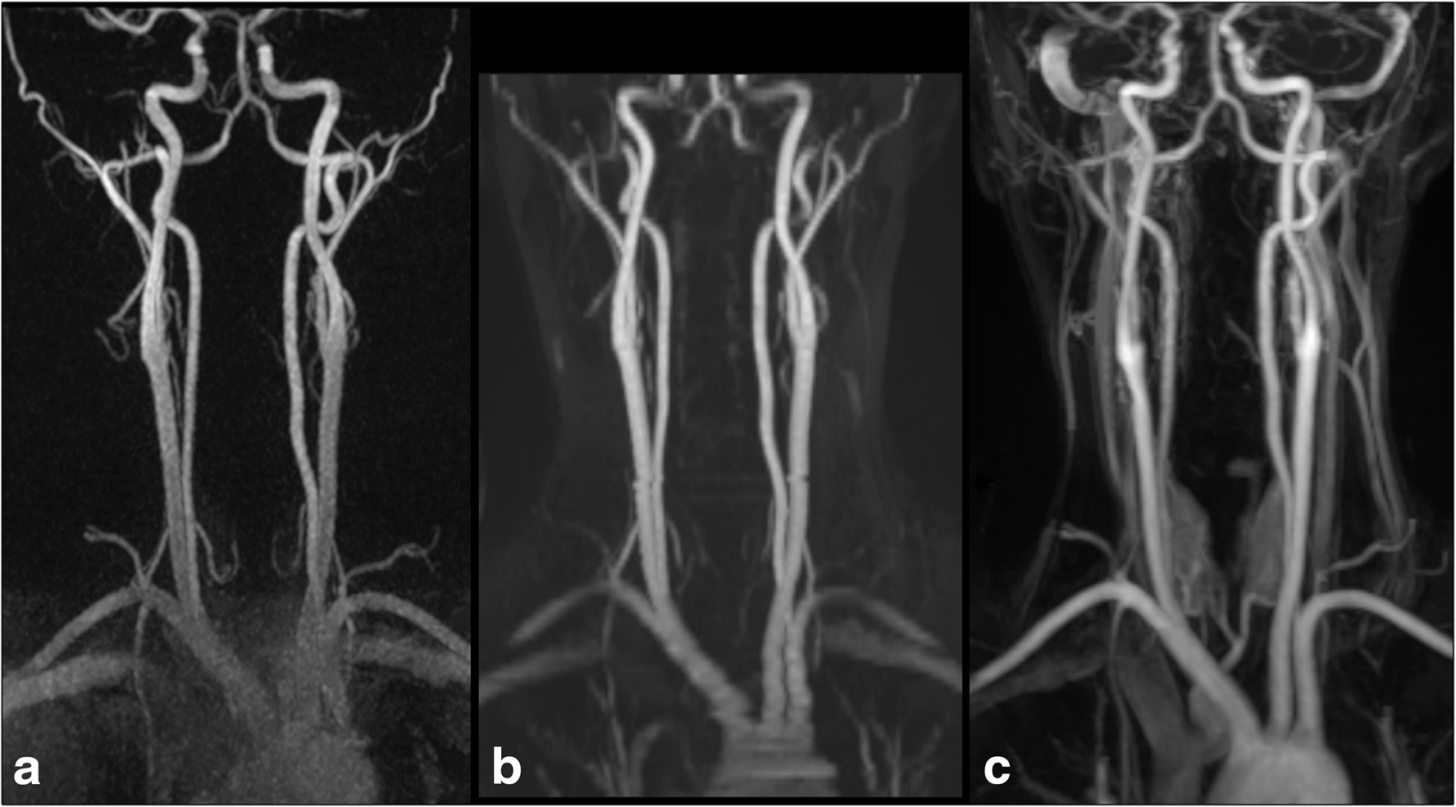
Representative coronal maximum intensity projection images obtained in a 42-year-old female with (**a**) nonenhanced hASL MRA, (**b**) nonenhanced TOF MRA and (**c**) CEMRA

**Fig. 4:**
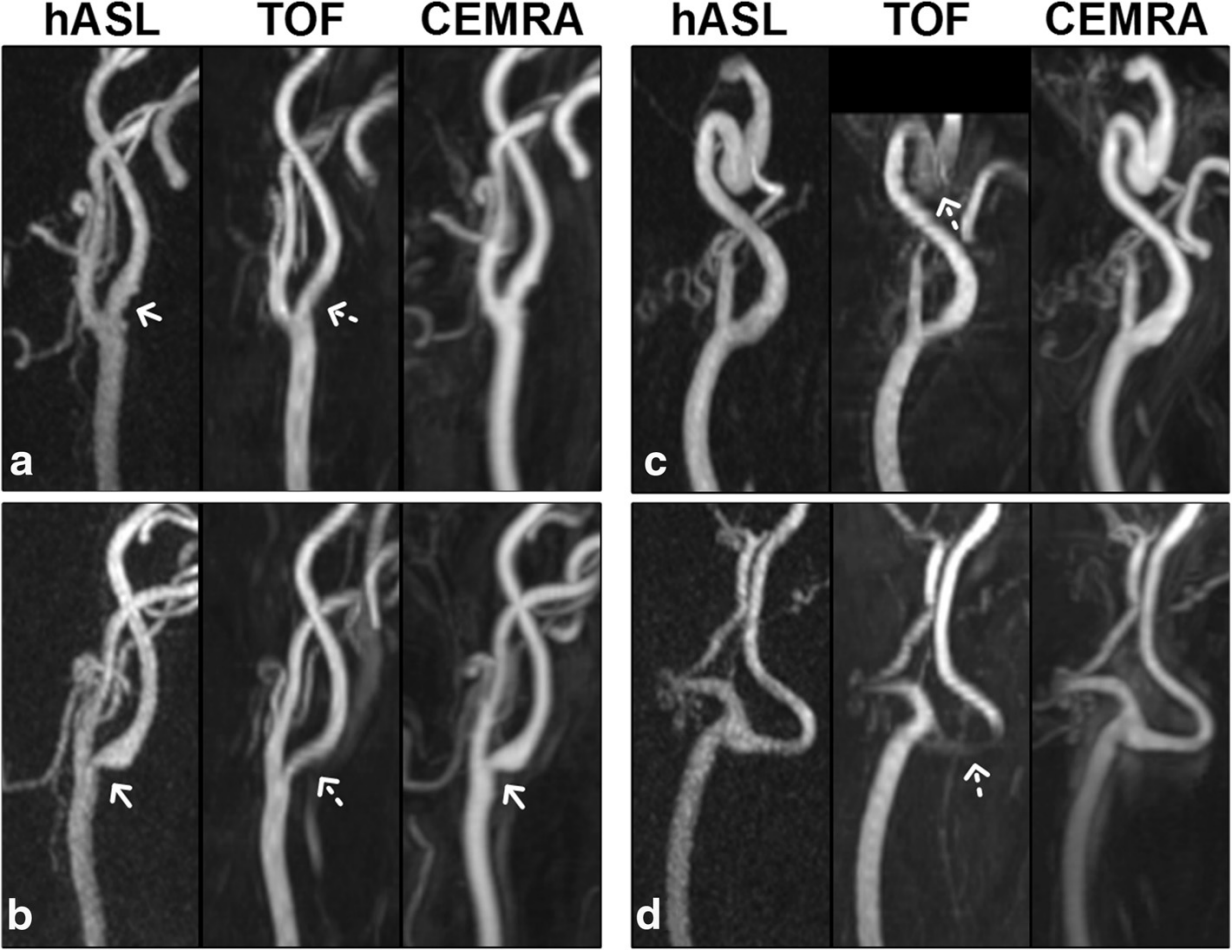
Representative maximum intensity projection images (30 mm thickness) of four carotid bifurcations obtained with hASL, TOF and CEMRA. **a** Luminal irregularity in the proximal internal carotid artery (ICA) of a 78-year-old male is well depicted by hASL (*arrow*), obscured by TOF (*dashed arrow*), and corroborated by CEMRA. **b** Moderate stenosis of the contralateral ICA in the same patient (*arrows*). Note the agreement between hASL and CEMRA in terms of arterial morphology and severity of disease; saturation of the carotid bulb, however, is evident with TOF (*dashed arrow*). Carotid bifurcations in (**c**) an 84-year-old female and (**d**) a 55-year-old male. Signal saturation effects observed with TOF (*dashed arrows*) are not observed with hASL. There is excellent correspondence of arterial morphology between hASL and CEMRA

**Fig. 5:**
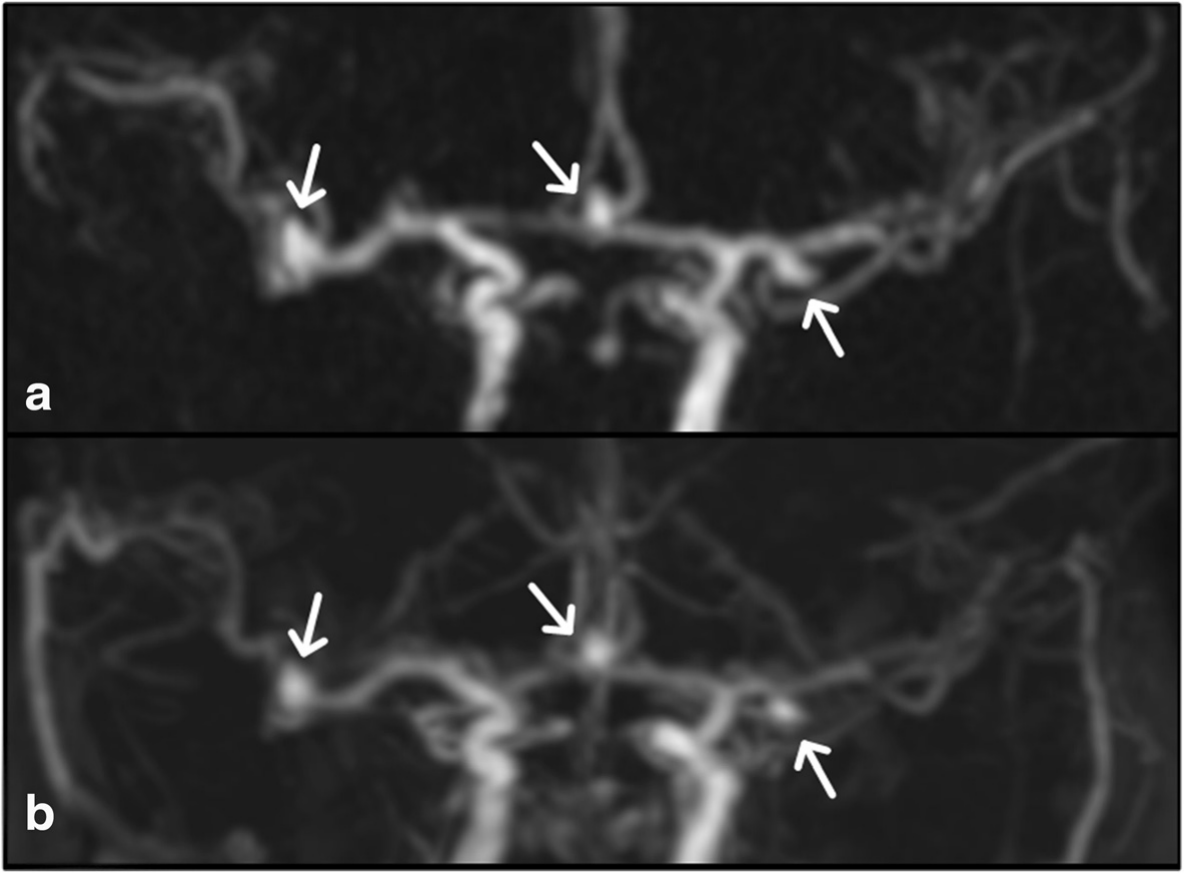
Coronal maximum intensity projection images of 65-year-old female with intracranial aneurysms (*arrows*) obtained with (**a**) nonenhanced hASL MRA and (**b**) CEMRA. Note the excellent depiction of the aneurysms and with hASL MRA and correspondence with CEMRA. TOF results not shown due to insufficient coverage

Representative angiograms obtained with hASL MRA at 3 Tesla are shown in Figs. 3 and 4. With hASL, the intracranial arteries were visualized if the field of view was sufficiently large in the head-foot direction (Fig. 5). Image quality scores and inter-rater agreement values for each technique with respect to location are summarized in Table 2. CEMRA provided significantly better image quality than the nonenhanced techniques across all arterial locations for both reviewers, except in the left and right carotid siphons (*P* < 0.05), where image quality between CEMRA and nonenhanced hASL MRA did not significantly differ for reviewer 2. When scores were aggregated across all locations, CEMRA provided the best image quality, with median scores of 4 for both reviewers (*P* < 0.001), followed by hASL MRA (median scores of 4 and 3 for reviewers 1 and 2) and 2D TOF (median scores of 3 and 2 for reviewers 1 and 2).

**Table 2:** Image Quality Scores and Inter-rater Agreement

	Image Quality								
	hASL		TOF		CEMRA		Inter-rater Agreement (AC1)		
Arterial Location	R1	R2	R1	R2	R1	R2	hASL	TOF	CEMRA
1. left CCA ^*^	2.0(2.7) ^**^	2.0(1.9)	2.0(2.3)	2.0(1.9)	4.0(4.0) ^***^	4.0(3.7) ^***^	0.39	0.37	0.71
2. right CCA ^*^	2.0(2.6)	2.0(2.1) ^**^	2.0(2.3)	2.0(1.8)	4.0(4.0) ^***^	4.0(3.8) ^***^	0.46	0.34	0.78
3. left bulb and prox. ICA ^*^	4.0(3.7) ^**^	4.0(3.4) ^**^	3.0(2.8)	3.0(2.6)	4.0(3.9) ^***^	4.0(3.9) ^***^	0.67	0.42	0.91
4. right bulb and prox. ICA ^*^	4.0(3.6) ^**^	4.0(3.5) ^**^	3.0(2.8)	3.0(2.6)	4.0(3.9) ^***^	4.0(3.9) ^***^	0.75	0.39	0.91
5. left mid-cervical ICA ^*^	4.0(3.6) ^**^	4.0(3.6) ^**^	3.0(2.9)	3.0(2.6)	4.0(4.0) ^***^	4.0(3.9) ^***^	0.71	0.38	0.94
6. right mid-cervical ICA ^*^	4.0(3.6) ^**^	4.0(3.6) ^**^	3.0(2.8)	3.0(2.6)	4.0(3.9) ^***^	4.0(3.9) ^***^	0.79	0.45	0.97
7. left petrous ICA ^*^	3.5(2.8) ^**^	4.0(3.4) ^**^	1.5(1.9)	2.0(1.7)	4.0(4.0) ^***^	4.0(4.0) ^**^	0.47	0.36	0.94
8. right petrous ICA ^*^	4.0(3.0)	4.0(3.4) ^**^	2.0(2.2)	2.0(1.8)	4.0(4.0) ^***^	4.0(4.0) ^**^	0.62	0.30	0.91
9. left ECA ^*^	4.0(3.5) ^**^	4.0(3.4) ^**^	3.0(2.6)	2.0(2.4)	4.0(3.9) ^***^	4.0(3.7) ^***^	0.47	0.56	0.81
10. right ECA ^*^	4.0(3.4) ^**^	4.0(3.4) ^**^	3.0(2.6)	2.0(2.5)	4.0(3.9) ^***^	4.0(3.8) ^***^	0.60	0.60	0.78
All Locations ^*^	4.0(3.3) ^**^	3.0(3.1) ^**^	3.0(2.6)	2.0(2.3)	4.0(3.9) ^***^	4.0(3.8) ^***^	0.61	0.43 ^****^	0.87 ^****^

Image quality data are presented as median (mean); 1: non-diagnostic, 4: excellent

Data summarize findings from locations depicted by all three techniques

*R1* reviewer 1, *R2* reviewer 2, *CCA* common carotid artery, *ICA* internal carotid artery, *ECA* external carotid artery

^*^ *P* < 0.05, Bonferroni-corrected Friedman test across techniques

^**^ *P* < 0.05 vs. TOF for the same reviewer

^***^ *P* < 0.05 vs. hASL and TOF for the same reviewer

^****^ *P* < 0.05 vs. hASL for AC1 value

For both reviewers, hASL MRA provided better image quality than TOF in the following 8 locations of the extracranial carotid arteries: bilateral proximal internal carotid arteries (ICAs), bilateral mid-cervical ICAs, bilateral petrous ICAs, and bilateral external carotid arteries (*P* < 0.05). Reviewers 1 and 2 scored the left and right common carotid arteries better with hASL than with TOF, respectively. For the intracranial arteries (which were not assessable by 2D TOF because of limited axial coverage), image quality scores for non-contrast hASL MRA (median/mean values of 2.0/2.4 and 2.0/2.5 for reviewers 1 and 2, respectively) and CEMRA (median/mean values of 3.0/2.7 and 3.0/2.9) significantly differed (*P* < 0.05) for reviewer 2 but not reviewer 1.

### Qualitative analysis: inter-rater agreement

Inter-rater agreement in the carotid arteries was substantial for hASL MRA (AC1 = 0.61, 95 % confidence interval (CI): 0.54–0.67; *P* < 0.001), moderate for TOF MRA (AC1 = 0.43, 95 % CI: 0.36–0.50; *P* < 0.001) and almost perfect for CEMRA (AC1 = 0.87, 95 % CI: 0.83–0.91; *P* < 0.001). In the intracranial arteries, inter-rater agreement was fair for both hASL MRA (AC1 = 0.60, *P* < 0.001) and CEMRA (AC1 = 0.55, *P* < 0.001).

### Qualitative analysis: impact of age and body mass index

There were no significant correlations between image quality and age for hASL (*ρ* = 0.08, *P* = 0.62), TOF (*ρ* = -0.31, *P* = 0.07) and CEMRA (*ρ* = -0.09, *P* = 0.59). Similarly, there were no significant correlations between image quality and body mass index for hASL (*ρ* =−0.06, *P* = 0.73), TOF (*ρ* = 0.01, *P* = 0.96) and CEMRA (*ρ* =−0.01, *P* = 0.95).

### Detection of arterial pathology

Using CEMRA as the reference standard, hASL MRA detected 5 of 5 instances of internal carotid arterial pathology (4 stenoses, 1 fibromuscular dysplasia) with no false positives, and 4 of 4 instances of intracranial arterial pathology (3 aneurysms, 1 middle cerebral artery stenosis) with no false positives. Due to limitations in image quality, only 2 of the 5 instances of carotid pathology (2 stenoses) were detected using TOF MRA; there were no false positive findings with TOF. Intracranial pathology was not evaluable by TOF due to insufficient coverage.

### Quantitative analysis

**Fig. 6:**
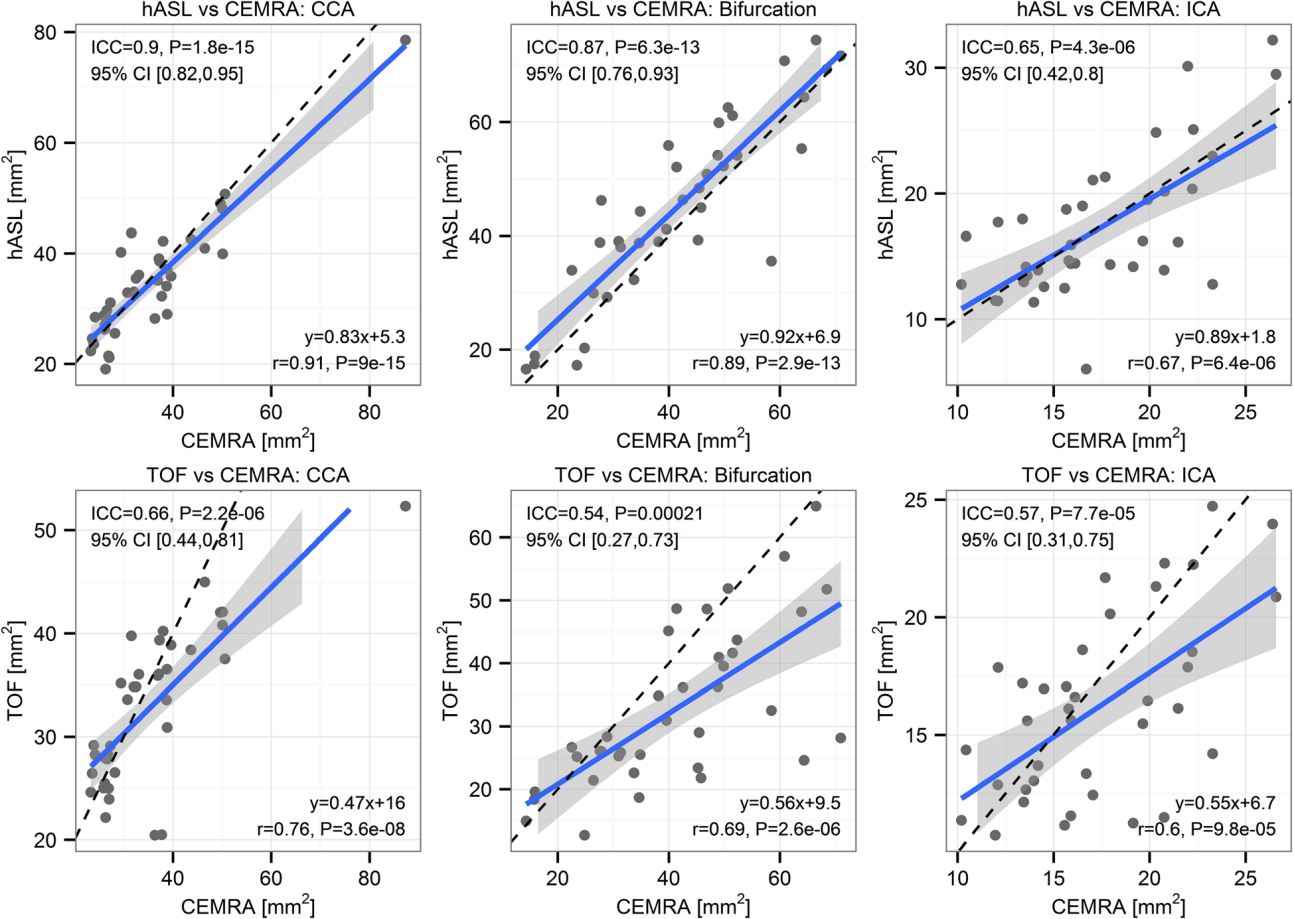
Scatter plots of cross-sectional lumen area of the common carotid artery (CCA) (*leftmost column*), carotid bifurcation (*middle column*), and internal carotid artery (ICA) (*rightmost column*). Compared with CEMRA, better agreement and correlation of cross-sectional lumen areas as assessed by intraclass correlation coefficient (ICC) and linear regression analysis, was observed with hASL MRA, as compared with TOF MRA. TOF MRA tended to underestimate luminal area as compared with CEMRA. Solid lines and gray areas show the lines of best fit and the 95 % confidence intervals, respectively. Linear regression equations are shown at bottom right. Dashed lines are lines of unity

Measurements of cross-sectional lumen area are summarized in Fig. 6. Compared to CEMRA, better agreement of cross-sectional lumen area was obtained with hASL MRA than with TOF at the common carotid artery (ICC = 0.90 for hASL versus 0.66 for TOF, *P* < 0.05), carotid bifurcation (ICC = 0.87 versus 0.53, *P* < 0.05), and internal carotid artery (ICC = 0.65 versus 0.57). Results of the Bland-Altman analyses are shown in Table 3. hASL MRA had smaller absolute biases and smaller to comparable 95 % limits of agreement for cross-sectional lumen area as compared with TOF MRA. The three techniques differed in arterial sharpness (*P* < 0.001). Arterial sharpness was best with hASL MRA (0.74 ± 0.12 mm^-1^) (*P* < 0.001 versus TOF and CEMRA), followed by TOF (0.63 ± 0.13 mm^-1^) and CEMRA (0.57 ± 0.10 mm^-1^), which did not statistically differ.

**Table 3:** Bland-Altman Analyses of Cross-Sectional Lumen Area with Respect to CEMRA

Technique	Location	Bias (mm^2^)	95 % Limits of agreement (mm^2^)
hASL	CCA	−0.8	(−10.7, 9.1)
TOF	CCA	−2.6	(−18.2, 13.0)
hASL	Bifurcation	3.5	(−11.2, 18.2)
TOF	Bifurcation	−8.6	(−31.0, 13.8)
hASL	ICA	0.2	(−8.5, 8.2)
TOF	ICA	−1.1	(−8.4, 6.1)

Nonenhanced technique minus CEMRA

*CCA* common carotid artery, *ICA* internal carotid artery

## Discussion

In this retrospective study, we investigated whether ungated hASL MRA using a FLASH readout and Cartesian k-sampling trajectory could faithfully display the extracranial carotid arteries at 3 Tesla without the use of contrast agents. Our results indicate the affirmative. Although inferior to CEMRA, image quality obtained with hASL MRA was found to be superior to 2D TOF for displaying the carotid bifurcation, internal carotid arteries and external carotid arteries. In addition, inter-rater agreement was improved with hASL MRA as compared with TOF MRA. Furthermore, compared with values obtained with TOF, measurements of cross-sectional arterial area obtained with hASL better agreed with values obtained from first-pass CEMRA. Finally, arterial sharpness provided by hASL MRA was improved with respect to TOF and CEMRA.

Of the three techniques available for comparison in this study, CEMRA provided the best image quality and inter-rater agreement in the extracranial carotid arteries. This finding is not unexpected given that CEMRA is a fast, reliable and accurate technique for evaluating the extracranial carotid arteries at 1.5 and 3 Tesla [29–34]. For patients in whom Gd-based contrast is contraindicated or when it is useful to save contrast for other purposes (such as the assessment of cerebral perfusion in patients with suspected stroke), however, nonenhanced MRA remains an important diagnostic option. Nonenhanced MRA is also useful backup in circumstances when CEMRA is mistimed with respect to first-pass of contrast bolus and there is insufficient delineation of the carotid arteries due to an early acquisition, or considerable venous contamination due to a late acquisition. Also, given the growing concern of Gd accumulation in the brain following contrast-enhanced CMR studies with unknown long-term consequences [35, 36], review of high-quality nonenhanced MRA may afford one the option of skipping CEMRA.

Nonenhanced 2D TOF is routinely used for depicting the extracranial carotid arteries prior to CEMRA and in patients who cannot receive Gd-based contrast agents. In this study, hASL MRA provided better image quality than 2D TOF MRA at the carotid bulb and in the internal and external carotid arteries. The improved image quality of hASL for depicting the carotid arteries relative to 2D TOF is consistent with prior reports at 1.5 Tesla using bSSFP readouts [20, 21], and is ascribed to the method’s elimination of background signal, reduced sensitivity to saturation of the CMR signal from in-plane and recirculating blood flow, and higher spatial resolution in the head-foot direction. These results are also consistent with an in-vitro study that found that hASL FLASH MRA more accurately portrayed carotid arterial morphology than 2D and 3D TOF over a wide range of physiological flow rates [22]. In this work, we did not compare hASL to 3D TOF, which more accurately displays stenoses of the carotid bifurcation than 2D TOF [10, 37]. Nevertheless, it would have been impractical to acquire 3D TOF spanning the entire length of the extracranial carotid arteries due to scan time considerations and respiratory motion in the upper chest.

To our knowledge, this is the first study reporting the feasibility of using ASL-based MRA for portraying the extracranial carotid arteries in consecutive patients imaged in a clinical environment. Prior works have reported the feasibility of ASL-based MRA for depicting the carotid arteries of non-consecutive patients imaged in a non-clinical research environment [16, 17, 20, 21]. The results of our study therefore indicate that hASL MRA of the extracranial carotid arteries is feasible and can be performed by CMR technologists without the need for special training or expertise.

Recent works have reported the following nonenhanced alternatives to TOF for the evaluation of the extracranial carotid arteries at 3 Tesla: inversion-recovery bSSFP [12, 13]; inversion-recovery fast spin-echo [14]; and QISS using a FLASH readout [15]. Direct comparisons of hASL MRA with these recent methods were outside the scope of this retrospective study. Nonetheless, the following statements can be made after careful examination of the literature. Compared with hASL MRA, inversion-recovery bSSFP methods are expected to provide reduced scan times and motion sensitivity, due to the lack of signal subtraction. On the other hand, inversion-recovery protocols only partially suppress signal from background tissues such as muscle, fat and cerebrospinal fluid. This is in stark contrast to hASL MRA which eliminates background signal and therefore allows for the creation of rotating MIP images similar to what is done with CEMRA, without requiring one to first remove background signal prior to MIP generation (of note, background signal was removed for all image sets in this study to facilitate the fairest comparisons, but such processing was not necessary for hASL MRA). Furthermore, the FLASH readout used with hASL MRA is less sensitive than bSSFP imaging to B0 and B1 inhomogeneity, which are worsened at 3 Tesla [38]. To date, cardiac-gated inversion-recovery fast spin-echo angiography has provided promising initial results in volunteers, but the approach has used larger voxel sizes of 3.5 mm^3^ (versus 1.0 mm^3^ voxels for hASL MRA) and has been unable to visualize intracranial arteries due to dephasing of the CMR signal at the skull base [14]. In comparison, intracranial vessels were depicted with fair to good image quality with hASL MRA in the present study.

We did not evaluate the thoracic inlet in this study because of the lack of respiratory motion compensation. The recently described approach of cardiac-gated QISS FLASH MRA [15], which uses tilted overlapping thin slices, is expected to provide for better display the thoracic inlet and carotid origins than hASL MRA. Conversely, drawbacks of QISS FLASH MRA include the need for cardiac gating and the slice resolution constraints associated with 2D imaging. With respect to prior implementations of hASL MRA at 1.5 Tesla that have used radial sampling trajectories and bSSFP readouts [21, 22], the presented variant of hASL MRA (which applies a Cartesian sampling trajectory and a FLASH readout) is simpler to implement and is less sensitive to artifacts from gradient timing errors and main magnetic field inhomogeneity, especially at 3 Tesla, the preferred field strength for clinical neurovascular MRA [39–41]. Finally, compared with variants using bSSFP readouts, the use of a FLASH readout is expected to improve the display of turbulent flow near severe carotid stenoses [22].

This study had some limitations. One limitation is that there was a relatively low incidence of carotid arterial pathology in our cohort (13.5 %, 5 of 37 patients), reflecting the breadth of patients with neurologic symptoms undergoing carotid MRA. Nonetheless, nonenhanced hASL MRA detected all instances of carotid disease observed at CEMRA without any false positives; by comparison, limitations in image quality for 2D TOF resulted in 3 false negative findings. Another limitation is the exclusion of 8 of 45 patients because of non-diagnostic image quality caused by motion artifact. Even though the proportion of diagnostic scans between the three techniques (hASL, TOF, CEMRA) did not reach statistical significance, all non-diagnostic MRA scans were nonenhanced. In our experience, motion-corrupted nonenhanced MRA can often be salvaged by reminding the patient to hold still and re-acquiring the scan; of note, re-acquisition was not attempted for any scan included in this retrospective study.

In the display of intracranial vessels, subtractive arterial spin-labeled MRA has previously demonstrated the ability to depict flow alterations, collateral flow patterns and arterio-venous malformations within the brain [19, 42–47]. The display of intracranial arteries and CEMRA-confirmed intracranial aneurysms and stenoses in our study is in general agreement with these prior works and suggests that hASL MRA at 3 Tesla using a FLASH readout may have a role in the simultaneous evaluation of extracranial and intracranial arteries in a single nonenhanced acquisition.

## Conclusions

hASL FLASH MRA offers an appealing alternative to 2D TOF MRA for nonenhanced MRA of the extracranial carotid arteries at 3 Tesla. The hASL FLASH MRA protocol may have utility as a pre-contrast scout, in the assessment of the carotid arteries in patients with renal insufficiency, and when it is desirable to save contrast agents for cerebral perfusion imaging.

## Abbreviations

ASL: Arterial spin labeling
bSSFP: balanced steady-state free precession
CI: confidence interval
CCA: common carotid artery
CEMRA: contrast-enhanced magnetic resonance angiography
CMR: cardiovascular magnetic resonance
ECA: external carotid artery
FLASH: fast low-angle shot
hASL: hybridized arterial spin labeling
ICA: internal carotid artery
ICC: intraclass correlation
MRA: magnetic resonance angiography
QISS: quiescent interval slice-selective
RF: radiofrequency
TOF: time of flight
